# Transradial approach for coronary angiography and percutaneos coronary intervention: personal experience

**DOI:** 10.1186/s43044-019-0006-2

**Published:** 2019-09-05

**Authors:** Jaafar Sadeq Aldoori, Ali Ibrahem Mohammed

**Affiliations:** 1 Department of Cardiology, Slemani Cardiac Hospital (SCH), Qanat street, Sulaymaniyah, Region of Kurdistan 46001 Iraq; 2 College of Medicine, University of Sulaimani, Sulaymaniyah, Region of Kurdistan Iraq

**Keywords:** Coronary angiography; Percutaneous coronary intervention; Transradial; Transfemoral; Radial artery occlusion

## Abstract

**Background:**

The transradial approach (TRA) has already become popular worldwide, but only recently has gained acceptance among Iraqi interventional cardiologists. The aim of this study is to document single operator experience with TRA and to test the benefit of assessing dual hand circulation before the TRA. It was an observational prospective study. Over a 2-year period (Jan 1, 2015, to Dec 31, 2016), 1561 patients underwent transradial coronary angiography (CAG) and/or percutaneous coronary intervention (PCI) by a single operator. Patients were divided into two groups: A (the first 450 patients), in which dual hand circulation was assessed by Allen’s test or plethysmography/oximetry test before TRA, and B (1111 patients) in which TRA was done without assessing dual hand circulation.

**Results:**

A total of 1561 patients were included, 69.1% males and 30.9% females. The mean age was (57 ± 10.0) years. We performed 1684 procedures (1005 CAG and 679 PCIs). The total transradial success rate was 95.6%, and PCI procedural success rate was 96.5%. The crossover rate from radial to femoral access was 4.4%. The primary causes for crossover were severe tortuosity of the aorta and brachiocephalic trunk, radial artery spasm, puncture failure, and radial loop. The main complication was radial artery occlusion (RAO) (3.7%). There were no cases of hand ischemia or complications that need surgical repair or blood transfusion. No statistically significant difference between groups A and B was observed regarding hand ischemia, the incidence of RAO, or the crossover rate.

**Conclusions:**

TRA is safe and can be applied in the majority of cases. The routine assessment of dual hand circulation before TRA might not be necessary.

## Background

Coronary angiography (CAG) and percutaneous coronary intervention (PCI) can be performed via the femoral, brachial, or radial arteries. The femoral approach had traditionally been the primary approach for most operators [1]. Following the first report of radial CAG by Campeau in 1989 and radial PCI by Kiemeneij et al. in 1993, there is an increase in use of transradial access around the world [2–4]. The major advantage of the TRA is the reduction in the incidence of complications related to the site of puncture associated with early ambulation, reduction in hospital stay, and consequently reduction in costs, making way for interventions in an outpatient care regimen [5–7]. The dual blood supply of the hand limits the potential for limb-threatening ischemia [8, 9]; therefore, assessment of dual hand circulation is considered essential before performing TRA.

Although TRA had been used as the preferred approach for CAG and PCI for more than two decades across the world, unfortunately, its use in Iraq is still limited. Only a few Iraqi cardiologists in few cardiac centers use the radial access as the default approach, while the majority of cardiologists still prefer the femoral access. In our center “Slemani Cardiac Hospital” (SCH), the TRA was started more than 6 years ago, and nowadays, more than 90% of coronary diagnostic and interventional procedures are done via the radial access. SCH is the first public cardiac center in Sulaymaniyah, Iraq.

The aim of this study is to document single operator experience with the TRA for CAG and PCI both in the elective and emergency setting, and to test the value of assessing dual hand circulation before the procedure.

## Patients and methods

It was an observational prospective study conducted in Slemani Cardiac Hospital (SCH). The study was approved by the “Scientific and Ethical Committee” of SCH in December 2014. Informed written consent to participate in the study was provided by all participants. Over a 2-year period (Jan 1, 2015, to Dec 31, 2016), 1561 patients were admitted to SCH and underwent transradial CAG and/or PCI by the same operator (the author).

To investigate the benefit of assessing dual hand circulation before the TRA, we divided the study population into two groups: groups A (the first 450 cases) and B (all other patients). In group A, dual hand circulation was assessed by Allen’s test or plethysmography/oximetry test and patients with abnormal tests were excluded from the study. While in group B, TRA was done without any assessment for dual hand circulation.

The patients were prepared for radial and femoral approaches. All the procedures were done through the right radial artery. Under local anesthesia (1–2 ml, Xylocaine 5%), radial punctures were performed using the transradial kit (Prelude, Merit Medical) which consisted of a 21-gauge needle, a 0.018″ guide-wire, and a short (7 cm long) sheath. Six-F sheath was used for all patients. After sheath insertion, a cocktail containing 200 μg nitroglycerin and 5000 IU unfractionated heparin (UFH) was injected into the radial artery.

For diagnostic CAG, the following catheters were used: 6F or 5F Tiger (TIG) catheter (Terumo, Japan) or 6F Ultimate catheter (Merit Medical) to cannulate both left and right coronary arteries or Judkin’s left (JL 6/3.5 and 6/4) and Judkin’s right (JR 6/4 and 6/3.5) catheters to cannulate the left and right coronary artery respectively.

For patients with PCI, Judkin’s guiding catheters (JL6/3.5 and JR 6/4) and extra back-up (EBU) guiding catheter (6/3.5) were the most widely used catheters for coronary engagement. All patients were loaded with dual antiplatelet drugs (300 mg aspirin and 600 mg clopidogrel for elective PCI, or 300 mg aspirin and 180 mg Ticagrelor for primary PCI). UFH (70_100 IU/kg) is the standard anticoagulation before the procedure. A drug-eluting stent (DES) (“Xience”, Abbott Vascular or “Resolute”, Medtronic) was used whenever stenting is indicated.

**Fig. 1:**
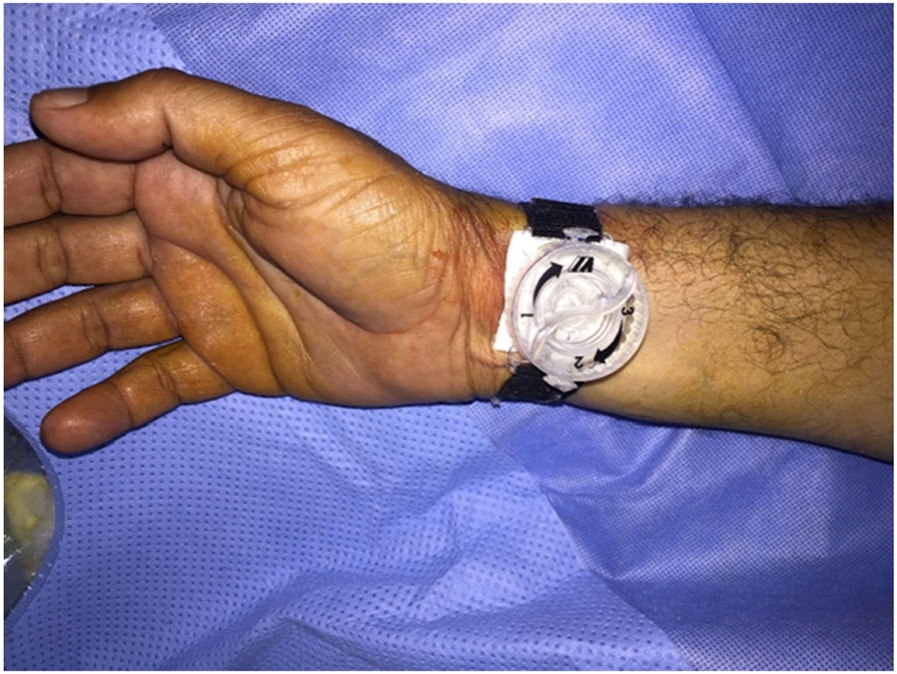
Finale radial compression device

The radial sheath was removed immediately after the procedure and compression was performed proximal to puncture site for 2 h using radial compression device (Finale; Merit medical) **(**Fig. 1**)**. Thereafter, a light pressure bandage was applied and removed in the next day.

Most of the elective PCI patients were discharged on the same day provided that no complications occurred in the first 6 h after the procedure. Patients with primary PCI were discharged after 48 h when they were stable. The site of radial puncture was examined before discharge and after 2 weeks, and radial artery patency was assessed by checking the radial pulse. Radial artery occlusion (RAO) was considered present in the absence of a radial pulse distal to the puncture site.

### Statistical method

Statistical analyses were performed using the SPSS 23. Continuous variables were analyzed and presented as mean ± SD whereas categorical variables were given as numbers (percentages). The comparison between categorical variables was done by chi-square test. *p* < 0.05 was considered statistically significant.

## Results

A total of 1561 consecutive patients were included. There were 1079 males (69.1%) and 482 females (30.9%). The age ranged from 26 to 95 years (mean of 57 ± 10.0). Baseline characteristics of the patients are summarized in Table 1.

**Table 1:** Baseline patients’ characteristics

	*N* (%)
Age/year	57 ± 10.0
Male	1079 (69.1)
Female	482 (30.9)
Hypertension	875 (56.1)
Diabetes	452 (29.0)
Smoking	691 (44.3)
Dyslipidemia	489 (31.3)

During the study period, we performed (1684) procedures, which include 1005 CAG (59.7%) and 679 PCI (40.3%). One hundred eighty-nine patients had elective PCI, 274 patients had CAG and PCI in the same session (ad Hoc PCI), 123 patients had second transradial PCI as part of staged procedure, and 93 patients underwent primary PCI (PPCI) for ST-elevation myocardial infarction (STEMI) (Table 2).

**Table 2:** Types of procedures

Procedure	*N* (%)
CAG	1005 (59.7%)
Total of PCI	679 (40.3%)
Elective PCI	189 (11.2%)
CAG + Ad Hoc PCI	274 (16.3%)
2^nd^ PCI	123 (7.3%)
PPCI	93 (5.5%)

Total transradial technical success rate was 95.6%, and PCI procedural success rate was 96.5%. The PCI procedures include single-vessel disease, multi-vessel disease, total occlusions, bifurcational lesions, left main stem (LMS) disease, and PPCI. Transradial PCI failed in 24 patients (3.5%), due to chronic total occlusion (CTO) in 22 patients and failure to cross the coronary lesion with balloon or stent in 2 patients.

Crossover from radial approach to femoral approach occurred in 69 (4.4%) patients. The main reasons for crossover were severe subclavian or aortic tortuosity in 16 (1.0%) patients, severe radial artery spasm which did not respond to multiple doses of intra-arterial nitroglycerin and IV analgesia in 14 (0.9%) patients, puncture failure in 11 (0.7%) patients, and radial loop in 9 (0.6%) patients. Other causes for crossover are shown in Table 3.

**Table 3:** Causes of crossover from radial to femoral approach

Cause of crossover	*N* (%)
Tortuous subclavian artery or aorta	16 (1.0%)
Radial artery spasm*	14 (0.9%)
Puncture failure	11 (0.7%)
Radial loop	9 (0.6%)
Small radial artery	4 (0.25%)
Subclavian artery occlusion	4 (0.25%)
Failure to engage the coronary arteries with guiding catheter	3 (0.2%)
Inadequate support of the guiding catheter	3 (0.2%)
Anatomic variations**	3 (0.2%)
Inability to cross the coronary lesion with balloon or stent	2 (0.1%)
Total	69 (4.4%)

*Which did not respond to multiple doses of intra-arterial nitroglycerin and IV analgesia

**Such as high origin of the radial artery, abnormal origin of the right subclavian artery, and abnormal right coronary artery origin

Crossover from radial to femoral access was higher in old-age patients (above 60 years) than younger patients (5.6% versus 3.5%); however, the difference between the two groups was statistically not significant (*p* value = 0.052) (Table 4).

**Table 4:** Relation of crossover to age group

		Patients with crossover	Total	*p* value*
Patient age	< 60	33 (3.5%)	922	0.052
	≥ 60	36 (5.6%)	639	
Total		69	1561	

*Significant level ≤ 0.05

The frequency of various complications was as follow: five patients (0.3%) had forearm hematoma which was treated conservatively. Radial artery dissection with extravasation of contrast in one patient (0.06%), which was resolved conservatively. Radial artery occlusion (RAO) was observed in 57 patients (3.7%). There were no cases of hand ischemia, pseudoaneurysm, arteriovenous fistula, or bleeding complications that need surgical repair or blood transfusions (Table 5).

**Table 5:** Types of complications

Complication	*N* (%)
Hematoma	5 (0.3%)
Radial artery dissection	1 (0.06%)
Hand ischemia	0 (0.0%)
Radial artery perforation	0 (0.0%)
Pseudoaneurysm	0 (0.0%)
Arteriovenous fistula	0 (0.0%)
Bleeding	0 (0.0%)
Radial artery occlusion (RAO)	57 (3.7%)

We found no statistically significant difference between groups A and group B regarding hand ischemia, the incidence of RAO, or crossover rate (*p* value = 0.052) (Table 6).

**Table 6:** Parameters according to assessment of dual hand circulation (group A, patients who had assessment of dual hand circulation before TRA; group B, patients who underwent TRA without assessing dual hand circulation)

Parameter		Group A	Group B
Number		450 (28.8%)	1111 (71.2%)
Gender	Male	307(68.2%)	772 (69.5%)
	Female	143(31.8%)	339 (30.5%)
Mean age/year		(56 ± 10.0)	(58 ± 10.0)
Procedure	CAG	404(89.8%)	601 (54.1%)
	PCI	29 (6.5%)	160 (14.4%)
	CAG+ ad hoc PCI	15 (3.3%)	259 (23.3%)
	PPCI	2 (0.4%)	91 (8.2%)
	2^nd^ PCI	20 (4.4%)	103 (9.3%)
Cross over into femoral		19 (4.2%)	50 (4.5%)
Complications	Hematoma	2 (0.4%)	3 (0.27%)
	Radial artery dissection	0	1 (0.09%)
	Bleeding	0	0
	Radial artery perforation	0	0
	Arteriovenous fistula	0	0
	Pseudoaneurysm	0	0
	RAO	16 (3.5%)	41(3.7%)

*p value = 0.052 (not significant)

## Discussion

The radial approach is an attractive alternative to the classical femoral approach for CAG and PCI. The radial artery is very superficial, making it easy to puncture, and bleeding is controlled by compression. There are no major nerves or veins near the radial artery, thus minimizing the risk of nerve and vascular injuries [10, 11]. The benefits of TRA have been documented in many studies. These benefits include less bleeding [10–17], lower morbidity, early ambulation, lower total hospital costs [10, 18], patient preference and comfort, same-day discharge is possible, less chance of developing ischemia due to dual blood supply of the hand, and easy access for the patients with myocardial infarction (MI) and aortic aneurysm [10, 19, 20]. The approach is advantageous for people with severe occlusive aortoiliac disease or difficulty lying down (e.g., due to back pain, obesity, or congestive heart failure) [8, 21].

As has been shown in several studies, the radial access permits treatment of the same type of patients and lesions as femoral access provides [11, 17, 22–27]. The radial artery readily accommodates 6-F sheaths, and sheathless 7-F techniques have recently been described [28, 29]. Thus, there is no limitation to performing complex PCI successfully via the radial approach [30]. High-risk subsets such as unprotected left main coronary artery [31], bifurcational lesions, and chronic total occlusions [32] can all be readily addressed through radial access [30]. Results from our study show that the transradial PCI was associated with high procedural success rates (96.5%) and favorable clinical outcomes in all patients, both in the elective and emergency (STEMI) setting.

Patients with STEMI are the most intensely anticoagulated, and many had received thrombolytic therapy prior to arrival at the PCI center, so they have high risk of bleeding. Thus, the potential for access-site complications is highest in this group and the potential benefit from TRA is greatest [33, 10, 34]. Bleeding after an acute myocardial infarction (MI) is associated with worse short- and long-term outcomes and prolonged hospitalization [35, 36]. Many trials have proved that TRA has lower risk of bleeding in STEMI patients as compared to transfemoral approach (TFA). The RIVAL (radial versus femoral access for coronary angiography and intervention in patients with acute coronary syndromes) study showed that TRA is associated not only with a lower rate of local vascular complications in the overall population, but also with a reduction in mortality in the setting of acute PCI [37, 38]. These results have been confirmed in another randomized study, the RIFLE-STEACS study (radial versus femoral randomized investigation in ST-segment elevation acute coronary syndrome). This trial specifically compared the TRA and the TFA for primary PCI, in which a relative reduction in access-site complications and in mortality of nearly 40% was found with TRA [38–40].

According to the latest (2018) European Society of Cardiology (ESC) guidelines on myocardial revascularization, the radial access should be the standard approach for coronary angiography and PCI in all clinical settings (class I, level of evidence A) [41].

Currently, it is well established that TRA nearly abolishes access-site complications in all patients. All studies comparing TRA versus TFA have demonstrated a reduction in major bleeding with TRA, both in the elective and the acute setting [38, 42, 43]. When access-site complications still occur after TRA, they usually have a benign course and do not influence the prognosis of patients [38]. Surgical intervention for the treatment of hematoma or arteriovenous fistulae has been rarely observed [5, 44]. The incidence of complications in our study matches the literature findings.

The advantages of TRA extend to the elderly patients as well. In a recent meta-analysis of 777,841 elderly patients by Alnasser et al [45], TRA compared to the TFA was associated with a significant reduction in vascular complications and stroke, but mortality benefit was seen only among patients presenting with STEMI.

Despite the aforementioned advantages, there are potential disadvantages to the TRA [8]. The TRA is technically more complex than the TFA due to the greater difficulty in cannulating the artery, the possibility of spasm, anatomical variations in the arteries of the upper limb, and the change in manipulation of the catheters that is necessary to cannulate the coronary arteries [11, 46, 47]. All these difficulties result in an increase in the length of procedural time and the need for a significant learning curve [8, 11, 47]. Some interventions may be technically challenging via the radial route due to the size of the technology required, e.g., large bore rotational atherectomy [8, 48]. Moreover, TRA is usually more demanding and needs longer procedural time in elderly patients because of the frequent presence of specific vascular abnormalities such as tortuosity, calcifications, or arterial loops [4].

TRA has been associated with a greater access crossover rate, which was reported to be 4–7% in various studies [4, 49, 50]. Louvard et al. reported a crossover rate of 10% in the first 50 cases, and 3–4% after other 500 cases; then, it stabilizes at less than 1% after 1000 procedures [1, 51]. In our study, the crossover rate was 4.4%, with higher rate in older patients (≥ 60 years old) than younger patients (5.6% versus 3.5% respectively). However, the difference between the two groups was statistically not significant. In the meta-analysis of elderly patients by Alnasser et al., access site crossover rate was higher for TRA compared to the TFA (11% vs. 3%, *p* = 0.0003), but remains acceptably low [45].

While serious bleeding complications are uncommon, the TRA bears the risk of radial artery occlusion (RAO). The incidence of RAO varies between 3 and 10%, according to different studies and protocols [40, 52, 53]. RAO rarely results in serious adverse events nor is it symptomatic, but the artery is lost for future procedures [33, 40, 54]. Therefore, any effort should be taken to reduce the risk of RAO [40].

The occurrence of RAO is determined by one or more of the following three factors, and all of them are operator dependent and therefore preventable: incomplete anticoagulation, catheter- artery mismatch, and prolonged arterial compression [38, 55]. The incidence of RAO is directly related to the ratio between the sheath and artery size [1, 55, 56]. Therefore, smaller guiding catheters are potentially advantageous leading to less arterial spasm, pain, and post-procedural RAO [1]. Prolonged and forceful post-procedure radial artery compression is perhaps the most common cause of RAO [33, 55]. Patent or non-occlusive artery hemostasis—that is, applying enough pressure to the radial access site to achieve hemostasis and yet maintaining antegrade flow in the radial artery—has been shown to drastically reduce the incidence of RAO [38, 40, 55].

In our study, the rate of RAO was 3.7%. Moreover, 123 patients had second TRA (as part of staged PCI) reflecting the preserved patency of the radial artery after TRA, or it may indicate that RAO is temporary in some cases. The use of 6F radial sheath in all procedures, followed by routine administration of heparin (5000 IU) into the radial artery after sheath insertion, and the controlled pressure over the radial artery with “radial compression device” all resulted in the reduction of the incidence of RAO.

Since the introduction of TRA, it has been recommended to assess dual hand circulation before use [57]. This assessment is done by using Allen’s test which is subjective, or the more objective oximetry/plethysmography test. In most TRA studies, patients with abnormal tests were excluded from the studies. In our study, the majority of patients (71.2%) underwent transradial procedure without any assessment of dual hand circulation (group B); however, this did not result in worse outcomes such as hand ischemia or higher rates of access crossover or RAO. In an international transradial practice survey by Bertrand et al. [57] which included 1107 interventional cardiologists from 75 countries, 23.4–30.8% of operators did not assess dual hand circulation at all. Because the Allen test or the oximetry/plethysmography test have not been shown to be predictive of hand ischemia in case of RAO, it remains uncertain whether the assessment of dual hand circulation before TRA is required [57].

## Conclusions

The TRA for CAG and PCIs is effective and safe and can be applied in the majority of cases. It dramatically reduces access site complications. The routine assessment of dual hand circulation before TRA might not be necessary, however more studies are needed to confirm our results.

## Data Availability

The datasets used and/or analyzed during the current study are available from the corresponding author on reasonable request.

## Abbreviations

CAG: Coronary angiography
CTO: Chronic total occlusion
DES: Drug-eluting stent
EBU: Extra back-up
ESC: European Society of Cardiology
F: French gauge
IU: International unit
IV: Intravenous
JL: Judkin’s left
JR: Judkin’s right
LMS: Left main stem
MI: Myocardial infarction
PCI: Percutaneous coronary intervention
PPCI: Primary percutaneous coronary intervention
RAO: Radial artery occlusion
SCH: Slemani Cardiac Hospital
SD: Standard deviation
SPSS: Statistical Package for the Social Sciences
STEMI: ST elevation myocardial infarction
TFA: Transfemoral approach
Tig: Tiger
TRA: Transradial approach
UFH: Unfractionated heparin
